# Nutritional Status of Orphaned and Separated Children and Adolescents Living in Community and Institutional Environments in Uasin Gishu County, Kenya

**DOI:** 10.1371/journal.pone.0070054

**Published:** 2013-07-26

**Authors:** Paula Braitstein, Samuel Ayaya, Winstone M. Nyandiko, Allan Kamanda, Julius Koech, Peter Gisore, Lukoye Atwoli, Rachel C. Vreeman, Corey Duefield, David O. Ayuku

**Affiliations:** 1 Indiana University School of Medicine, Department of Medicine, Indianapolis, Indiana, United States of America; 2 Moi University College of Health Sciences, School of Medicine, Department of Medicine, Eldoret, Kenya; 3 University of Toronto, Dalla Lana School of Public Health, Toronto, Canada; 4 Moi University College of Health Sciences, School of Medicine, Department of Child Health and Pediatrics, Eldoret, Kenya; 5 Moi Teaching and Referral Hospital, Eldoret, Kenya; 6 Academic Model Providing Access to Healthcare (AMPATH), Eldoret, Kenya; 7 Moi University College of Health Sciences, School of Medicine, Department of Mental Health, Eldoret, Kenya; 8 Indiana University School of Medicine, Department of Pediatrics, Indianapolis, Indiana, United States of America; 9 Brown University, School of Public Health, Center for Statistical Sciences, Providence, Rhode Island, United States of America; 10 Moi University College of Health Sciences, School of Medicine, Department of Behavioral Sciences, Eldoret, Kenya; Wadsworth Center, United States of America

## Abstract

**Objective:**

To describe the nutritional status of orphaned and separated children and adolescents (OSCA) living in households in the community (HH), on the street, and those in institutional environments in western Kenya.

**Methods:**

The study enrolled OSCA from 300 randomly selected households (HH), 19 Charitable Children’s Institutions (CCIs), and 100 street-involved children. Measures of malnutrition were standardized with Z-scores using World Health Organization criteria; Z-scores ≤-2 standard deviations (sd) were moderate-severe malnutrition. Data were analyzed using multivariable logistic regression adjusting for child age, sex, HIV status, whether the child had been hospitalized in the previous year, time living with current guardian, and intra-household clustering for adequacy of diet and moderate-severe malnutrition.

**Results:**

Included are data from 2862 participants (1337 in CCI’s, 1425 in HH’s, and 100 street youth). The population was 46% female with median age at enrolment of 11.1 years. Only 4.4% of households and institutions reported household food security; 93% of children in HH reported an adequate diet vs. 95% in CCI’s and 99% among street youth. After adjustment, OSCA in HH were less likely to have an adequate diet compared to those in CCI’s (AOR 0.4, 95% CI 0.2–1.0). After adjustment, there were no differences between the categories of children on weight-for-age, weight-for-height, or BMI-for-age. Children living in HH (AOR 2.6, 95% CI: 2.0–3.4) and street youth (AOR: 5.9, 95% CI: 3.6–9.5) were more likely than children in CCI’s to be low height-for-age.

**Conclusion:**

OSCA in HH are less likely to have an adequate diet compared to children in CCI’s. They and street children are more likely to be moderately-severely low height-for-age compared to children in CCI’s, suggesting chronic malnutrition among them.

## Introduction

UNICEF estimates that as of 2010 there were 153 million orphaned children and adolescents living in the world [1]. While 13% of the world’s children under the age of 18 years live in sub-Saharan Africa, 36% of the world’s orphans live in the region [1]. Approximately 27% of these orphans were orphaned due to AIDS [1], [2]. Communities and families in sub-Saharan Africa have been faced with a growing challenge of providing care to these vulnerable children. Over 90% of all orphans not living with a surviving parent are cared for by extended families [3].

With 46% of Kenyans living below the poverty line [4], combined with the sheer numbers of children requiring care and support, many families struggle to meet traditional care-taking expectations and responsibilities [5], [6], [7], [8], [9], [10]. As of 2004, 17% of households with children in sub-Saharan Africa were taking care of an average of 1.8 orphans [8]. Grandmothers are the primary caretakers for approximately half of all orphaned children [8]. Extended families, particularly those headed by grandparents, are not always able to meet the material needs of the orphaned children in their care [5], [10], [11], [12]. Many caretakers are not capable of providing care simply because of ill health or old age [10]. Several studies have found that orphaned children lived disproportionately in the poorest households, and are more likely to be underweight compared to their non-orphaned counterparts [13], [14], [15], [16].

While the extended family is considered the ideal environment for taking care of orphaned children, a child’s relatives may be unwilling or unable to adequately support them, either because of cultural issues [10] or competing financial obligations (for example with one’s own biological children). What is not clear is whether orphaned children living in other types of environments fare better. Institutions have traditionally been seen as places of last resort, with organizations such as UNICEF and others taking a clear position about the role of institutional environments in caring for orphaned children: “This strategy is not a viable solution.” [17]. Many countries including Rwanda, Uganda, and Ethiopia are adopting national de-institutionalization policies [17], [18].

We sought to understand whether, in this region of western Kenya, orphaned and separated children living in the community were in fact better off in terms of their nutritional status compared to children living in institutional environments. The primary objective of this analysis was to describe the baseline nutritional status of orphaned and separated children aged 0–18 years living in a random sample of households in the community compared to children living in institutional environments, compared to a small non-random sample of children living ‘on’ or ‘of’ the street [19].

## Methods

### Study Setting

Uasin Gishu (UG) County is one of the 47 counties of Kenya, with its headquarters in Eldoret, about 375 kilometers northwest of Kenya’s capital city, Nairobi. Major industries include textiles, wheat, pyrethrum and corn and the region is considered highly agriculturally productive. In 2010, UG County had approximately 894,179 individuals from 202,291 households, of whom 41.5% were aged 14 years or less [20]. The majority of the UG County population (61.4%) reside in rural settings [21] in comparison to 67.7% of the population in the rest of Kenya and 77.3% in East Africa [22]. Approximately 51.3% of the population in UG County live below the Kenyan poverty line [21]. Eldoret has a total population of 289,389 and is currently the 5th largest city in the country [21]. It is home to Moi University, Moi Teaching and Referral Hospital, and AMPATH-Plus, a large HIV care program [23].

### OSCAR’s Health and Well-Being Project

The Orphaned and Separated Children’s Assessments Related to their (OSCAR’s) Health and Well-Being Project is a 5-year longitudinal cohort study evaluating the effects of different care environments on the physical and mental health outcomes of orphaned and separated children aged 18 years of age or less. The study intends to describe these care environments, determine whether they are able to meet the basic socioeconomic needs of the resident children, and examine the effect of care environment on resident children’s physical and mental health over time. The study began enrolling participants in June 2010. The project follows a cohort of children from 300 households, 19 Charitable Children’s Institutions (CCIs) (orphanages, rescue centers, etc.), and 100 street-involved children and youth in UG.

### Human Subjects Protection

This study was approved by the Moi University College of Health Sciences and Moi Teaching and Referral Hospital Institutional Research and Ethics Committee and the Indiana University Institutional Review Board. Written informed consent was provided by the head of household, Director of CCI, or in the case of the street youth, by the District Children’s Officer (DCO). Individual written informed assent was provided by each child aged 7 years and above. Fingerprints were used for both children and guardians who were unable to sign or write their name. Verbal assent was provided by children under 7 years but old enough to have a basic understanding of the study purpose and study procedures. The consent and assent processes were documented through the use of consent and assent notes.

### Study Population: Eligibility, Sampling and Recruitment

#### Community-based Households

The project aimed to randomly sample 300 households within eight locations representing families caring for orphaned and separated children in the UG County. In order to obtain a representative sample of households caring for orphans in UG County, the project utilized three sampling arms: cash-transfer (CT) households, non-cash transfer households from the same sub-Location (SSL), and non-cash transfer households from a different sub-Location (DSL). The CT program is a government social support initiative that provides regular and predictable (unconditional) cash transfers to poor households taking care of orphans and vulnerable children. The main objective of the CT-OVC program is to encourage fostering and retention of OVC within their families and communities as well as to enhance their human capital development [24]. The CT program targets sub-locations that are the most socioeconomically deprived. Sub-Locations are administrative boundaries within locations and are headed by an Assistant Chief. 100 households were sampled from each category (CT, SSL, DSL) and weighted to reflect the number of households required per location based on the population, to ensure appropriate distribution.

For non-CT households, Assistant Chiefs and Village Elders drew up lists of all the households in their villages and sub-Locations caring for orphaned and/or separated children. The lists contained the names of the head of household, their national ID number where available, telephone number where available, the village in which they live, the number of children in the household, and the number of orphaned children in the household. These lists became the sampling frame for the random selection of non-CT (SSL and DSL) households to invite as per the sampling strategy just described.

The DCO oversees the government CT program and provided the study lists of households receiving the government subsidy in each location. These lists were used for simple random sampling for the CT households.

In order not to ‘single out’ the orphaned child in the household, all children in the household were eligible to participate. Consenting, registration, enrolment and all individual study procedures for recruited households took place at the central OSCAR clinic located at Moi Teaching and Referral Hospital (MTRH) in Eldoret.

#### Charitable Children’s Institutions (CCIs)

Under the Kenyan Children Act (2001), orphanages and other institutions serving orphans are called CCI’s (i.e. children’s homes) if they are able to accommodate ≥20 children [25]. All such institutions being subject to the Kenyan Children Act (2001), located within UG county boundaries, were eligible for recruitment into the study. The UG County Children’s Department maintains a list of registered and unregistered institutions, and has monthly meetings with them in the UG Children’s Services Forum. Two methods were used to identify and recruit CCI’s to participate in the project. First the project utilized the lists of registered CCI’s maintained by the UG Children’s Department and contacted them with a formal letter of introduction from the DCO. Secondly, snowball sampling techniques were used with community members and other stakeholders to identify and contact non-registered CCI’s. The OSCAR project became a member of the UG Children’s Services Forum and was given the opportunity to discuss the research project with forum members. Support was sought from the forum members and the project hoped to identify and sample all eligible CCI’s. The CCI’s were instrumental in identifying names and locations of other CCI’s to the project that could be approached and introduced to the project. In total, there were 21 eligible CCI’s identified in the UG County through the two strategies that the project wanted to recruit. For those not able to attend the Forum meeting, we arranged individual meetings with them and/or their Boards of Directors to discuss the study. Of 21 identified eligible CCI’s in the UG County that were contacted, 20 agreed to participate and one declined. The project arranged appointments to visit the 20 CCI’s that agreed to participate to facilitate enrolment and assessments of children on their premises. All study procedures for the children in CCI’s took place *in situ* at the institution.

#### Street youth

Street youth were recruited directly from the street as well as through community-based organizations using snowball sampling methods by a trained and experienced street youth outreach worker. Children and youth aged ≤18 years of age willing to participate were referred to the central OSCAR clinic for assenting and registration. All study procedures for the street youth took place at the central clinic.

A more detailed description of the project recruitment and sampling procedures is available elsewhere [26].

### Definitions

A single orphan was defined as a child whose mother *or* father was deceased, and a double orphan as one for whom parents were both deceased. A separated child was defined as one for whom at least one parent is completely absent from the child’s life [27]. UNICEF defines children living and working on the street in three categories: *children of the street*, *children on the street* and *children from street families* [*19*]. *Children on the street* spend a portion of their time on the street, working to provide an economic contribution to their family; however, they often return home at night to sleep, maintaining familial ties. *Children of the street* both work and sleep on the streets and have an absence of regular contact with family members. *Children from street families* live with their families in the street. Normal nutritional status was defined as a Z-score of-1 standard deviations (sd) to+2 sd. Z-scores greater than or equal to+2 sd were defined as over-nutrition. Mild malnutrition was a Z-score of-1 sd to-2 sd. Moderate to severe malnutrition was defined as a Z-score less than or equal to-2 sd.

### Sources of Data

Sociodemographic and clinical characteristics were assessed and documented through a standardized clinical encounter form and process. The clinical encounter was intended to be an enhanced well-child ‘check-up’ including a complete history and physical review of systems and symptoms. Household or institutional level data including household food security were collected *in situ* by trained Community Health Workers or the Project Manager respectively, and validated through random household audits.

#### Household food security and adequacy of diet

Adequacy of diet in terms of quantity and quality was assessed by the project nurse using the individual level component of the Household Food Insecurity Access Scale (HFIAS) specifically adapted by the USAID Food and Nutrition Technical Assistance (FANTA) project for use in Developing Countries [28]. Household Food Security was assessed during the site assessment using the household level component of HFIAS.

#### Anthropometric measurements

Weight was measured using a digital or infant weighing scale depending on the age of the child and their ability to stand unassisted. It was measured to the nearest 0.1 kg with the child lightly clothed. Height was taken using a measuring instrument. For infants, an infant heightometer was used. For others, the standing body height was measured to the nearest 0.1 cm. The participants stood (without shoes) on a horizontal platform with heels together. They were asked to draw themselves to full height without raising the shoulders, with hands and arms hanging relaxed, and with the feet flat on the ground.

#### HIV status

HIV testing was conducted on all participants aged at least 18 months using rapid fingerstick testing by nationally certified HIV counselors. Children aged less than 18 months were referred to AMPATH for HIV DNA testing.

#### Symptoms of tuberculosis

During the clinical encounter, participants were screened for tuberculosis using the modified Kenneth-Jones scoring system [29], [30], [31]. These criteria were consistent with the tuberculosis case definition for children under 15 years recommended by the World Health Organization (WHO) [32].

### Statistical Analysis

The primary outcomes for this analysis were household food security, adequacy of diet, and nutritional status. Nutritional status was based on Z-scores calculated using World Health Organization macros in SAS v. 9.3 for weight-for-height or length (0–5 years), weight-for-age (0–10 years), height- or length-for-age (0–18 years), and body mass index (BMI)-for-age (6–18 years).

The analysis was restricted to baseline data collected June 2010-March 2012. Parametric and non-parametric descriptive statistics were employed to summarize both categorical and continuous variables. For continuous variables, mean and median together with standard deviation and inter-quartile range were calculated, respectively. The Chi-Square test was used to test for associations between categorical/dichotomous variables. The Fisher’s exact test was also used if some cells had expected value of less than 5. Missing values for selected covariates are reported. The HFIAS score is a continuous measure of the degree of food insecurity in the household over the previous 30 days. A score is calculated for each household by summing the coded frequency of experience for each question. The maximum score for a household is 27, the minimum is 0. The higher the score, the more food insecurity the household experienced. These scores were also categorized into 4 categories of food insecurity: none, low, moderate, severe [28].

A logistic regression model was created to examine the probability of household food insecurity among households in the community compared to CCI’s, adjusting for guardian age and education. Models were also created to examine adequacy of diet, and moderately or severely low height-for-age, weight-for-age, weight-for-height, and BMI-for-age in the different care environments. The number of street children aged less than 10 years was small, so street youth were not included in the models of weight-for-height or weight-for-age. The reference category for each model was children living in CCI’s. In these models we adjusted for potential confounding factors: child age, sex, HIV status, whether the child was hospitalized in the previous year, time living with present guardian, and intra-household or institutional clustering. For the latter we used robust standard errors.

Sensitivity analyses were conducted. First, non-orphaned/non-separated children living with both biological parents were excluded. In the second, we excluded two CCI’s which were atypical compared to the others. These were: a Rescue Shelter for street youth, supported partially by government and partially by the Catholic Church, and a government probation detention centre for youth with nowhere else to go for their probation.

All analyses were conducted using SAS Version 9.3.

## Results

Included in this analysis were data from children living in 300 households, 19 Charitable Children’s Institutions (CCI’s), and 100 street youth. In total, 2862 children were enrolled and had baseline assessments done: 1337 in CCI’s, 1425 living in households in the community (HH’s), and 100 street youth).

### Socio-demographic and Clinical Characteristics

Overall, the population was 46% female with a median age at enrolment of 11.1 years (interquartile range, IQR: 6.84–14.41). There were differences in the proportions of children by gender among the three categories of care environment: 51% of children living in households were male compared to 55% of children in CCI’s and 77% of street youth. Street youth were also on average the oldest with a median age of 15.3 years, compared to 11.3 years in CCI children, and 10.5 years among children in HH. Among the HH children, 53.1% and 22.1% were single and double orphaned or separated respectively; compared to 35.3% and 52.3% in CCI’s and 35% and 49% of street youth; 24.8%, 12.1% and 16% of children in HH, CCI, and street youth respectively had both parents alive and were living with them (Table 1).

**Table 1:** Baseline sociodemographic and clinical characteristics of children in Charitable Children’s Institutions (CCI’s), community-based households (HH), and street youth participating in the Orphaned and Separated Children’s Assessments Related to their (OSCAR’s) Health and Well-Being Study.

Characteristics	Overall	CCI’s	HH	Street Youth
	N = 2862 (%)	N = 1337 (%)	N = 1425 (%)	N = 100 (%)
Gender				
** Male**	1540 (53.8%)	740 (55.4%)	723 (50.7%)	77 (77.0%)
** Female**	1322 (46.2%)	597 (44.6%)	702 (49.3%)	23 (23.0%)
Missing		**–**	**–**	**–**
**Age in years (median, IQR** * **)**	11.1 (6.84–14.41)	11.3 (6.6–14.4)	10.5 (6.7–14.1)	15.3(12.3–17.3)
Missing	–	–	–	–
**Orphan status**				
Single orphan or separated	1263 (44.1%)	472 (35.3%)	756 (53.1%)	35 (35.0%)
Double orphan and separated	1063 (37.1%)	699 (52.3%)	315 (22.1%)	49 (49.0%)
Both parents alive & child is living with them	531 (18.6%)	162 (12.1%)	353 (24.8%)	16 (16.0%)
Missing	5 (0.2%)	4 (0.3%)	1 (0.07%)	0 (0.0%)
**HIV-positive**	48 (1.7%)	28 (2.1%)	19 (1.3%)	1 (1.0%)
Missing	208 (7.3%)	79 (5.9%)	122 (8.6%)	7 (7.0%)
**Hospitalized in past 1 year**				
Yes	113 (3.9%)	73 (5.5%)	36 (2.5%)	4 (4.0%)
No	2652 (92.7%)	1194 (89.3%)	1366 (95.9%)	92 (92.0%)
Missing	97 (3.4%)	70 (5.2%)	23 (1.6%)	4 (4.0%)
**Symptoms of tuberculosis**				
Yes	82 (2.9%)	35 (2.6%)	46 (3.2%)	1 (1.0%)
No	2647 (92.5%)	1184 (88.6%)	1366 (95.9%)	97 (97.0%)
Missing	133 (4.6%)	118 (8.8%)	13 (0.9%)	2 (2.0%)
**Quality of diet**				
Adequate	2096 (94.2%)	1273 (95.2%)	1324 (92.9%)	99 (99.0%)
Inadequate	148 (5.2%)	54 (4.0%)	93 (6.5%)	1 (1%)
Missing	18 (0.6%)	10 (0.7%)	8 (0.6%)	0 (0.0%)
**Median weight(IQR)**	30 (20–44)	31.0 (20.0–46.0)	28.0 (20.0–43.0)	39.2 (33.0–46.5)

* IQR: Interquartile range.

The overall prevalence of HIV in these children and adolescents was 2.1% in CCI’s, 1.3% among children in households, and 1% (n = 1) street youth (p<0.001). Three percent of children in both CCI’s and HH had symptoms of tuberculosis, compared to 1 street youth (p = 0.426). Among children from CCI’s, 6% had been hospitalized in the previous year compared to 3% of children in HH and 4% of street youth (p<0.001).

### Household Food Security

Table 2 details the findings related to household food security. Households in the community scored much higher for food insecurity on the HFIAS index: 13.22 vs. 3.16 among the CCI’s (mean score difference p<0.001). Only 4.4% of households and institutions overall reported being food secure: 42% of CCI’s and 2% of HH (Table 3). Adjusted for guardian age, HHs were much more likely to have any household food insecurity compared to CCIs (OR: 35.6, 95% CI: 10.5–120.4).

**Table 2:** Detailed responses to and culminating scores of household food security*among Charitable Children’s Institutions (CCI’s) and community-based households (HH) participating in OSCAR’s Health and Well-Being Study.

In the past 30 days, did:	CCI	HH	P-value
	n = 19 (%)	n = 300 (%)	
**You worry that the household (HH) would not have enough food?**			
*** Sometimes/often***	5 (26.3**%**)	223 (74.3**%**)	**<0.0001** 1
*** Never/rarely***	14 (73.8**%**)	77 (25.7**%**)	
**Was any HH member not able to eat the kinds of foods they preferred because of a lack of resources?**			
*** Sometimes/often***	2 (10.5**%**)	221 (73.7**%**)	**<0.0001** 2
*** Never/rarely***	17 (89.5**%**)	79 (26.3**%**)	
**Did any HH member eat just a few kinds of food day after day due to a lack of resources?**			
*** Sometimes/often***	4 (21.1**%**)	210 (70.0**%**)	**<0.0001** 2
*** Never/rarely***	15 (78.9**%**)	90 (30.0**%**)	
**Did any HH member eat food that they preferred not to eat because of a lack of resources to obtain other types of food?**			
*** Sometimes/often***	3 (15.8**%**)	181 (60.3**%**)	**0.0001** 2
*** Never/rarely***	16 (84.2**%**)	119 (39.7**%**)	
**Did any HH member eat a smaller meal than you felt the child needed because there was not enough food?**			
*** Sometimes/often***	4 (21.0**%**)	193 (64.3**%**)	**0.0002** 2
*** Never/rarely***	15 (79.0**%**)	107 (35.7**%**)	
**Did any household member eat fewer meals in a day because there was not enough food?**			
*** Sometimes/often***	3 (15.8)	191 (63.7)	**<0.0001** 2
*** Never/rarely***	16 (84.2**%**)	109 (36.3**%**)	
**Was there ever no food at all in your household because there were not resources to get more?**			
*** Sometimes/often***	3 (15.8**%**)	85 (28.3**%**)	**0.2978** 2
*** Never/rarely***	16 (84.2**%**)	215 (71.7**%**)	
**Did any HH member go to sleep at night hungry because there was not enough food**			
*** Sometimes/often***	1 (5.3**%**)	58 (19.3**%**)	**0.2180** 2
*** Never/rarely***	18 (94.7**%**)	242 (80.7**%**)	
**Did any HH member go a whole day without eating anything because there was not enough food?**			
*** Sometimes/often***	0 (0.0**%**)	62 (20.7**%**)	**0.0309** 2
*** Never/rarely***	19 (100.0**%**)	238 (79.3**%**)	
**Overall Score:**	**3.16**	**13.22**	**<0.0001**

* Household food security as measured by the Household Food Insecurity Access Scale (HFIAS) specifically adapted by the USAID Food and Nutrition Technical Assistance (FANTA) project for use in Developing Countries [28].

1 Chi-square test.

2 Fisher exact test.

**Table 3:** Categorization of household food security*into food secure, mildly, moderately, or severely food insecure among Charitable Children’s Institutions (CCI’s) and community-based households (HH’s) participating in OSCAR’s Health and Well-Being Study.

		*Household type*		
Insecurity categories	Overall n, (%)	CCI, n (%)	HH, n (%)	P-value
Food secure	12 (4.4)	8 (42.1)	6 (2.0)	
Mildly food insecure	236 (74.0)	11 (57.9)	225 (75.0)	
Moderately foodinsecure	68 (21.3)	0 (0.0)	68 (22.7)	
Severe food insecure	1 (0.3)	0 (0.0)	1 (0.3)	
**Total**	**301 (100.0)**	**19 (100.0)**	**300 (100.0)**	**<0.0001** 1

* Household food security as measured by the Household Food Insecurity Access Scale (HFIAS) specifically adapted by the USAID Food and Nutrition Technical Assistance (FANTA) project for use in Developing Countries [28].

1 Fisher’s exact test.

### Adequacy of diet

In spite of widespread perceived household food insecurity, 93% of children in HH reported an adequate diet (compared to 95% in CCI’s and 99% of street youth, p = 0.009) (Table 1). In adjusted analysis including the whole cohort, there was no statistical association to indicate that either street youth or children in HH were more or less likely to have an adequate diet compared to children in CCI’s (Table 4). In sensitivity analysis, however, when children living with both biological parents were excluded and when atypical CCI’s were excluded, the orphaned and separated children in HH became statistically significantly less likely to have an adequate diet compared to their counterparts in CCI’s (AOR 0.4, 0.2–1.0) (Table 4).

**Table 4:** Unadjusted and adjusted*odds ratios (OR) and 95% confidence intervals (CI) describing moderate to severely low malnutrition (Z scores ≤-2 standard deviations) among orphaned and separated children aged 0–18 years living in Charitable Children’s Institutions (CCI’s), community-based households (HH), and those living on the street participating in OSCAR’s Health and Well-Being Study.

*Status at baseline:*	Unadjusted OR (95%CI)	Overall Adjusted* OR (95%CI)	Sensitivity 1– Orphaned/separated only	Sensitivity 2– Exclusion of atypical CCI’s
**Adequate Diet (vs. inadequate)**	n = 2839	n = 2688	n = 2172	n = 2541
Children in HH	0.60 (0.24–1.54)	0.55 (0.23–1.33)	**0.38 (0.15–0.96)**	**0.34 (0.12–0.96)**
Street youth	4.20 (0.49–36.07)	3.70 (0.41–33.15)	2.99 (0.34–26.74)	1.98 (0.22–18.12)
Children in CCI’s	1	1	1	1
**Moderate-severely low weight-for-height** 1	n = 380	n = 354	n = 257	n = 332
Children in HH	1.02 (0.55–1.90)	0.82 (0.51–1.33)	1.37 (0.43–4.36)	2.10 (0.83–5.35)
Children in CCI’s	1	1	1	1
**Moderate-severely low weight-for-age** 2	n = 1213	n = 1144	n = 863	n = 1091
Children in HH	0.87 (0.56–1.34)	1.02 (0.62–1.67)	0.96 (0.57–1.62)	1.13 (0.70–1.82)
Children in CCI’s	1	1	1	1
**Moderate-severely low height-for-age** 3	n = 2842	n = 2689	n = 2173	n = 2540
Children in HH	**2.27 (1.74–2.94)**	**2.62 (2.01–3.43)**	**2.74 (2.07–3.65)**	**2.68 (2.02–3.56)**
Street youth	**4.95 (3.13–7.82)**	**5.85 (3.59–9.53)**	**5.97 (3.64–9.79)**	**5.83 (3.55–9.57)**
Children in CCI’s	1	1	1	1
**Moderate-severely low BMI-for-age** 4	n = 2374	n = 2255	n = 1856	n = 2135
Children in HH	0.70 (0.49–1.01)	0.72 (0.51–1.02)	0.71 (0.49–1.03)	0.75 (0.52–1.09)
Street youth	0.58 (0.31–1.08)	0.62 (0.32–1.19)	0.65 (0.34–1.26)	0.65 (0.34–1.27)
Children in CCI’s	1	1	1	1

* Odds ratios are adjusted for intra-household/institution clustering, as well as child age, sex, HIV status, having been hospitalized in the previous year, and length of time with present guardian.

1 Among children 5 years and under.

2 Among children 10 years and under.

3 Among children 0–18 years of age.

4 Among children 10–18 years of age.

### Nutritional Status

**Figure 1:**
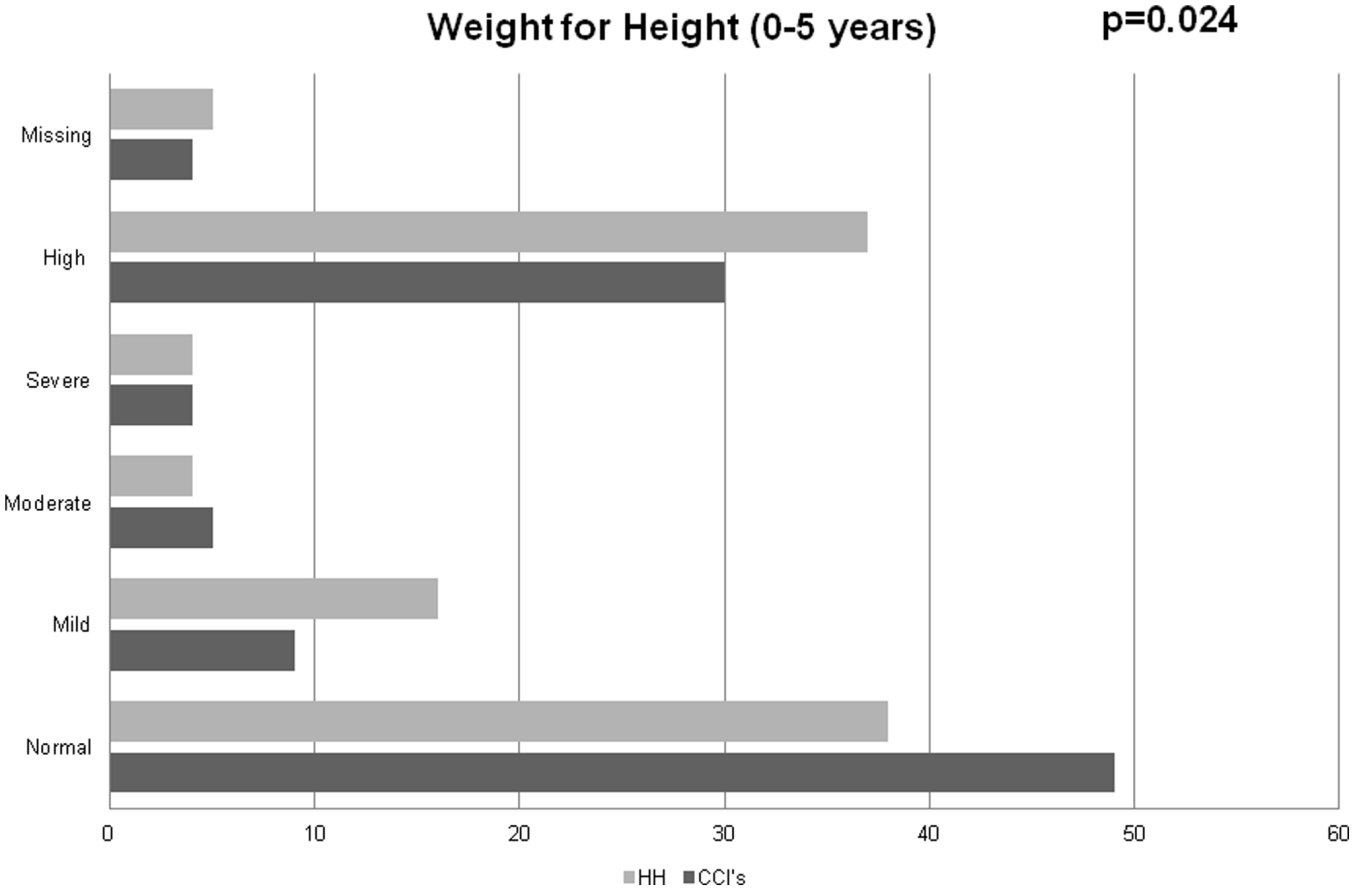
Weight-for-height among orphaned and separated children aged 0–5 years living in Charitable Children’s Institutions (CCI’s), in households in the community (HH) participating in OSCAR’s Health and Well-Being Study.

**Figure 2:**
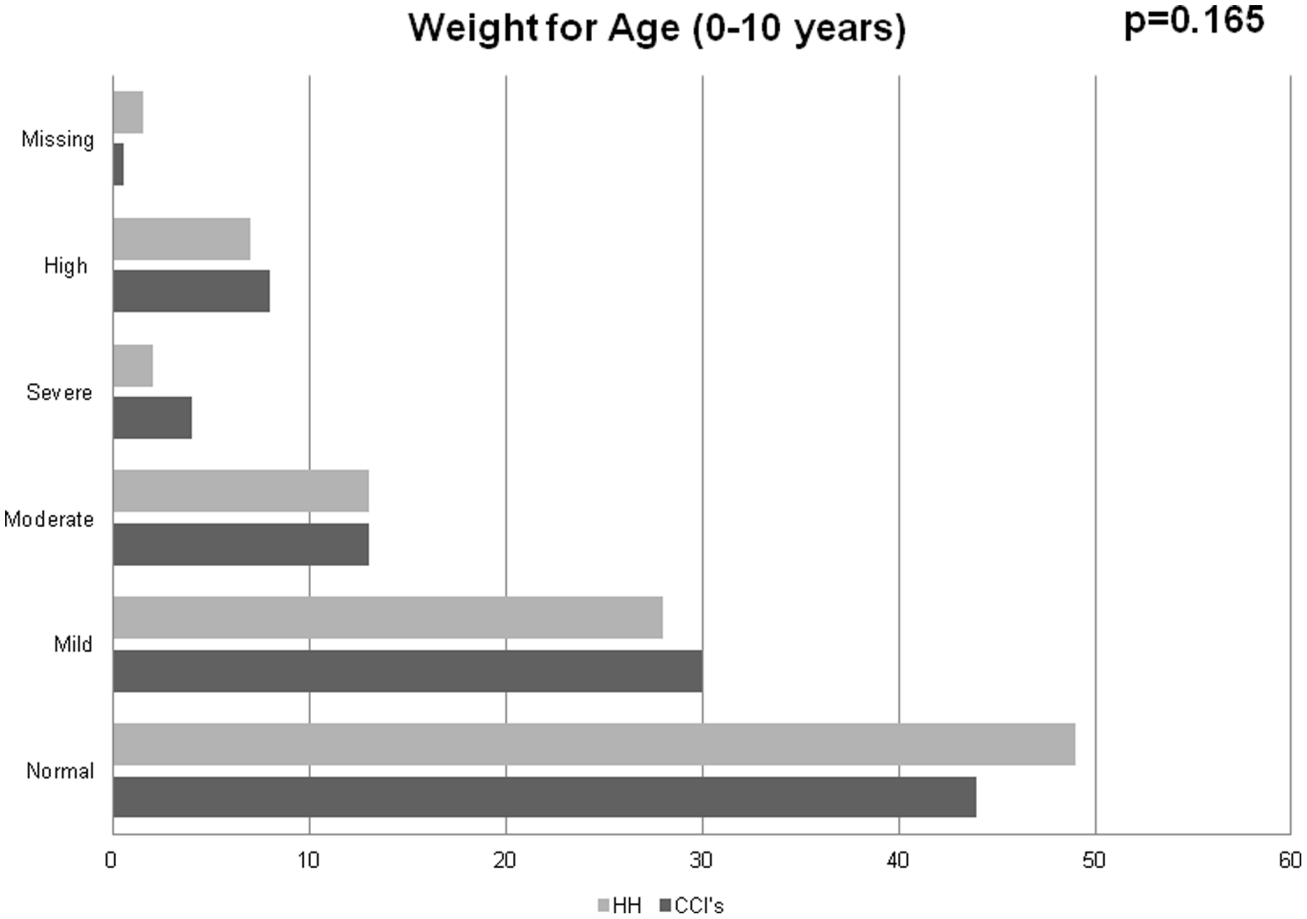
Weight-for-age among orphaned and separated children aged 0–10 years living in Charitable Children’s Institutions (CCI’s), in households in the community (HH) participating in OSCAR’s Health and Well-Being Study.

**Figure 3:**
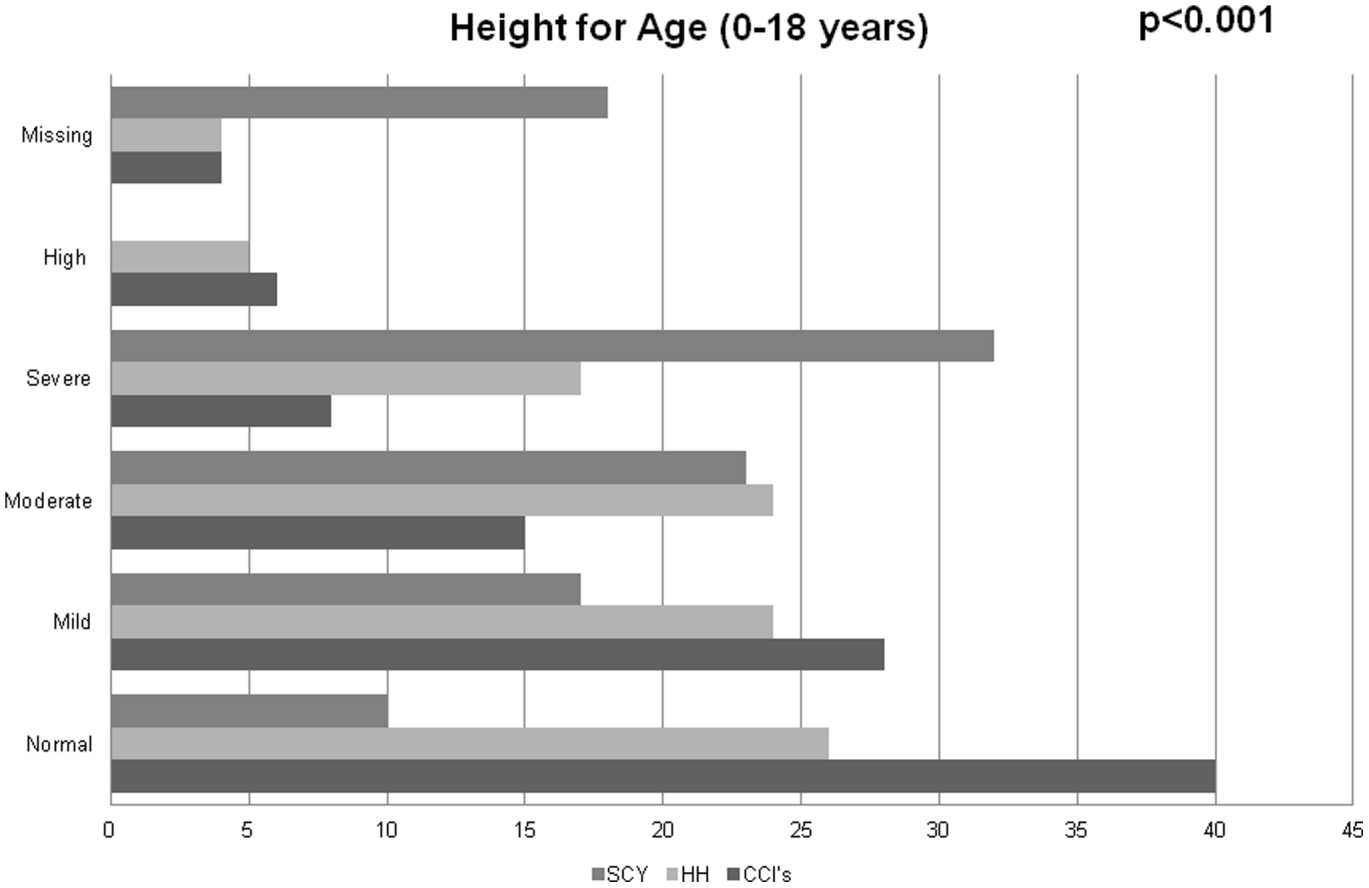
Height-for-age among orphaned and separated children aged 0–18 years living in Charitable Children’s Institutions (CCI’s), in households in the community (HH), and on the street (SCY) participating in OSCAR’s Health and Well-Being Study.

**Figure 4:**
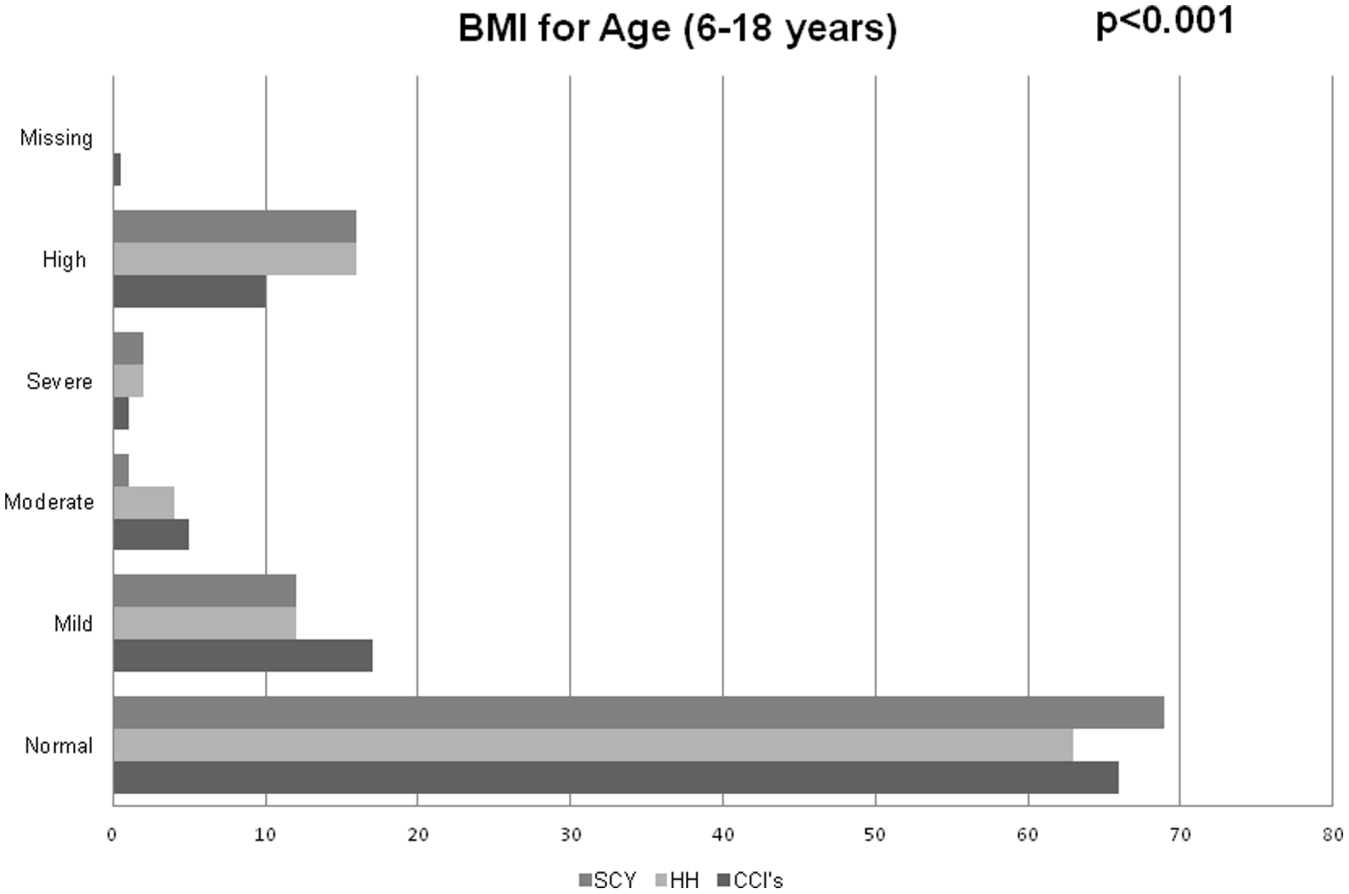
Body mass index (BMI)-for-age among orphaned and separated children aged 10–18 years living in Charitable Children’s Institutions (CCI’s), in households in the community (HH), and on the street (SCY) participating in OSCAR’s Health and Well-Being Study.

Unadjusted types of nutritional status between the different categories of children are summarized in Figures 1–4. Among 0–5 year olds, children in CCI’s were more likely to be normal weight-for-height compared to children in the households (p = 0.024) (Figure 1). Among 6–18 year olds, while there were no major differences in the proportions of children in CCI’s, households, and street youth with normal BMI-for-age (∼65%), there were higher proportions of both street youth and children in households with high BMI-for-age compared to children in CCI’s (19% and 16% vs. 10%, p<0.001) (Figure 1).

Stunting was widespread in this population, with 59% of all the children being abnormally low height-for-age (59% of children in CCI’s, 74% in households, and 88% of street youth) (p<0.001). Among 0–10 year olds, there were few differences in weight-for-age between households and institutions (street children were excluded because they were mostly older than 10 years) (Figures 1–4).

Table 4 details the unadjusted and adjusted odds ratios and 95% confidence intervals of nutritional status comparing the three categories of children. As described in the methods, the table includes two sensitivity analyses, first excluding children living with both biological parents, and then also excluding two CCI’s that were not typical local orphanages. Analyses were adjusted for age, sex, length of time with present guardian, HIV status of the child, hospitalization in the past year, and intra-household/institutional clustering. After adjusting for these factors, there were no statistical differences between the categories of children in terms of weight-for-age, weight-for-height, or BMI-for-age. However, children living in HH’s were more than twice as likely as children in CCI’s to have height stunting (low height-for-age) (AOR: 2.6, 95% CI: 2.0–3.4). Street youth were nearly 6 times more likely to have height stunting compared to children in CCI’s: (AOR: 5.9, 95% CI: 3.6–9.5). Sensitivity analyses did not affect these results.

## Discussion

There are several important and inter-related findings arising from this study. The first is that perceived food insecurity was extremely common in this cohort – this in a region with high agricultural productivity. Second, although the majority of children in HH and children in CCI had an adequate diet, we found that the orphaned and separated children living in HH’s were significantly less likely to have an adequate diet compared to children in CCI’s. This supports reports of intra-household discrimination against orphans within HH’s in the community since it was only seen in the sub-analysis excluding the non-orphans [33], [34]. Our third important finding is that stunting is extremely widespread, with an average of 74% of children in the study being stunted for their age according to WHO standards. Orphaned and separated children living in community households are 2–3 times more likely to have height stunting for their age compared to children living in CCI’s, suggesting chronic under- or malnutrition among children living in the community. Moreover, street youth are roughly 6 times more likely than children in CCI’s to be stunted, indicating that street youth have most likely experienced even more chronic malnutrition at least in their pasts. These data strongly support other evidence suggesting that 80% of children turn to street life because of extreme poverty or neglect in the home [35], [36], [37]. Our fourth key finding is that, in spite of widespread stunting among children in HH and street youth, 15–20% actually had high BMI-for-age, suggesting another kind of malnutrition increasingly evident in developing countries: over-weight and obesity [38], [39], [40].

These findings are supported by a variety of literature. It has previously been demonstrated that household food insecurity is increased among orphans living in households [41], and that orphans are more vulnerable to food insecurity than non-orphans [42]. It has also been demonstrated that orphaned children in sub-Saharan Africa tend to have more malnutrition compared to non-orphans [13], [15]. One study of institutionalized children and orphans living in households found that only 55% of children had at least three meals a day [43]. Stunting is a common phenomenon in sub-Saharan Africa and other developing world settings, both among orphans [44],[45] and non-orphans [46]. In sub-Saharan Africa, 38% of children under 5 years are estimated to be stunted for their age [47]. Our finding that 74% of the children and adolescents in our cohort are stunted likely exceeds the other reports because it is primarily a cohort of orphaned and separated children and the average age is 10 years. Our data suggesting that household food insecurity does not always translate into acute malnutrition is supported by another study from Kenya with similar findings [42], and requires more research. This may be explained by reporting bias because the measure of food insecurity used is based on perception by the head of the household rather than an empiric or objective measure of food insecurity. Thus while they may worry that their household doesn’t have enough to eat, they appear to be adequately feeding a majority of the children nevertheless.

Our finding that children living in CCI’s have improved nutritional status compared to orphaned children living in the community are supported by another study comparing orphaned children living in institutional and community-based settings in 6 geographically defined regions of 5 countries in Africa and Asia [48]. They found no differences in either height-for-age or BMI-for-age among children aged 6–12 years in the two environments and concluded that there was no evidence to support the hypothesis that orphaned children living in institutionalized settings would systematically have worse health outcomes compared to children in community-based settings. Our data indicate that within our East African setting, orphaned children in institutions are actually *more* likely to have an adequate diet and much *less* likely to have height stunting for their age compared to orphaned children in the community.

There are several strengths to our findings. The first is that these data come from a well-defined geographical area, and are representative of orphaned and separated children and adolescents in Uasin Gishu County, Kenya. This has allowed direct comparisons between children living in different care environments, within the same context of having reasonably well-structured child protection mechanisms in place. This reduces the likelihood of any bias arising from differential formulation or enforcement of local and national laws, policies, and regulation related to child welfare in previous studies [49]. The second is our relatively large sample size, which increases our statistical power to detect differences, and allow for important sensitivity analyses. A third strength of our study is that it covers children and adolescents from 0–18 years, in contrast to most other studies related to child nutrition which typically only involve children under 5 or under 10 years of age. This has enabled a comprehensive assessment of nutritional status, both acute and chronic, using age-appropriate measures in children *and adolescents*. Fourth, we have examined the issue of nutritional status within the context of household food security, and adequacy of diet, using standardized, validated tools, enabling a more holistic view of nutritional profile of these vulnerable children.

There may also be limitations to these findings. First, although the data on household food security were collected *in situ* by trained interviewers, the data are self-reported by the head of household. As such, they should be interpreted with caution because they measure perceived household food insecurity, rather than actual household food insecurity. Similarly, adequacy of diet is also self-report and is subject to reporting and recall bias. For example, guardians of young children in households may have been more likely to report better dietary intake if they were concerned about being perceived to not be taking good care of an orphaned child. In spite of training and supervision, the data on heights and weights may be subject to some non-differential measurement error.

In conclusion, these data have several important implications for understanding the optimal care environments for orphaned children and youth. While it would be ideal if supportive, extended family care was available for all orphaned and separated children and adolescents, those living in institutional environments in this region appear to have better food security, adequacy of diet and nutritional status compared to orphaned children in households in the community. This does not suggest that orphaned children should all be placed in institutional environments, but highlights the struggle for households and extended families to provide adequate food and nutritional support for orphans and the opportunity for strengthening community-based supports. The widespread stunting of growth among street children supports a history of food insecurity in their previous households. It is possible that increasing food and nutrition support to vulnerable families in the community would reduce the flow of children to the streets. More research is needed to determine how these differences change over time and to identify opportunities for intervention to improve the food security and care of orphaned and separated children in the community. Institutional environments play an important and necessary role in the care of orphaned and separated children and, at least in this setting, provide a benchmark against which community performance should be measured.
